# The lived experiences of clinical nurse managers regarding moral distress

**Published:** 2018-09-26

**Authors:** Alireza Nikbakht Nasrabadi, Mitra Khoobi, Mohammad Ali Cheraghi, Soodabeh Joolaei, Majid Ahmadi Hedayat

**Affiliations:** 1 *Professor, School of Nursing and Midwifery, Tehran University of Medical Sciences, Tehran, Iran. *; 2 *PhD Candidate of Nursing Education, Medical-Surgical Group, School of Nursing and Midwifery, Tehran University of Medical Sciences, Tehran, Iran. *; 3 * Professor, School of Nursing and Midwifery, Tehran University of Medical Sciences, Tehran, Iran.*; 4 *Professor, School of Nursing and Midwifery, Nursing Care Research Center, Iran University of Medical Sciences, Tehran, Iran.*; 5 *MSc, Research Deputy of Applied Sciences University, Ibn Sina Hospital, Tehran, Iran* *.*

**Keywords:** *Nurse manager*, *Lived experience*, *Moral distress*, *Desperation syndrome*, *Psycho-emotional trauma*

## Abstract

Ethical management with minimum moral distress is one of the main duties of nurse managers. There is no doubt that a better understanding of the experiences of nurse managers in morally challenging situations could have an effective role in improving health care systems. The present study aimed to investigate the lived experiences of clinical nurse managers regarding moral distress.

This hermeneutic phenomenological qualitative research involved the use of semi-structured interviews with nurse managers. The interviews were transcribed and analyzed by the Diekelman, Allen and Tanner approach. For this purpose, a total of 14 Iranian nurse managers with at least five years of experience in nursing management in hospitals were purposefully selected.

The findings related to nurse managers’ experiences of moral distress contained two main themes (psycho-emotional trauma and professional desperation syndrome) and four sub-themes (shame, emotional dissociation, helplessness, impaired professional identity).

The findings of the study indicated that in order to understand the phenomenon of moral distress among nurse managers, it is essential to investigate the moral distress experienced by them. We also found that although they experience moral distress in their daily decisions repeatedly, they are not fully aware of this phenomenon

According to the results, for clinical nurse managers, moral distress is an ambiguous situation like suspension along with uncertainty, fear and so on. They believed that experiencing this kind of conscious mistake is the reason for the occurrence of professional desperation syndrome and psycho-emotional trauma.

## Introduction

Moral distress is a kind of response by human beings to a situation in which the individual is prevented from performing or following-up a right, moral act. In general, moral distress is a psychosomatic phenomenon that would cause a feeling of discomfort and dissatisfaction in the individual at any time (1). Since moral issues are intertwined with the nursing profession, moral distress has become a serious concern for nurses (2). The Canadian Nurses Association has defined moral distress as having painful emotions in a situation where the correct and ethical act is known but cannot be performed (3). Therefore, when experiencing moral distress, individuals act against their personal and professional principles (1, 2, 4 - 6). Although moral distress is a part of the conditions governing human beings, nurses’ experiences of moral distress were first addressed about 5 or 6 decades ago (7, 8).

The importance of moral distress in nursing practice has been mentioned in various studies and has been discussed in the American Nurses Association and North American Nursing Diagnosis Association (6, 9). Most of the international and national studies in the field of moral distress have been performed on clinical nurses, especially those working in critical care units. The focus of most of these studies has been on moral distress in the context of poor inter-professional communication, professional ethical environment, physician-nurse relationship, and nurse-patient relationship (10 - 15). However, there are about 2.5 million nurses in America, and about 10% of them (250,000 nurses) hold managerial positions (16). In Iran the number of employed nurses is between 140,000 and 150,000, and about 10 percent of them (14,000 nurses) are managers in public and private sectors (17, 18).

Strategically, nurse managers have a unique position in the health system as their actions, behaviors and decisions have the greatest impact on their work environment and the profession in general (19, 20). A managerial work environment free from moral distress affects not only the managers, but the personnel as well (21, 22). Clinical nurse managers in these environments should play the role of managers, facilitators, supervisors and advocates. The latter role pertains to caregivers and therapists (physicians and nurses), clients (patients and their families), and organizations (health organizations or centers, and the executive and supportive management body). In addition to all these duties, nurse managers should consider resource allocation in accordance with their organizational policies and rules. Under the circumstance, role duality, moral distress and tension will occur as a result of an imbalance between the needs and values of the organization, nurses, patients and their families. 

Respecting moral standards for decision-making is associated with tension in every field (22 - 25). Ethical management with minimum moral distress is the main duty of nurse managers (26). Since managers’ performance is one of the effective factors in the improvement of efficiency, effectiveness and eventually organizational productivity, the efficiency of nurse managers is of utmost importance in the success of health systems (25).

It is clear that managerial levels affect all stratums of care. Therefore, nurse managers require an environment free from tension and distress so that they can achieve effective care outcomes and job satisfaction along with the rest of the medical team (27, 28).

In the reviewed national and international articles published between 2005 and 2016, no comprehensive report was found on nurse managers’ experiences of moral distress. However, some studies have shown that when clinical nurse managers encounter moral challenges and conflicts, their responsiveness to their organization, colleagues, and patients and their families will be affected (15). A review of former research produced numerous studies with a focus on clinical nurses during recent years. It seems essential, however, to conduct studies on nurse managers’ moral distress due to the sensitivity of their job and constant involvement with unplanned or even routine decisions (23). Therefore, a better understanding of the experiences of nurse managers in the field of moral distress could greatly improve occupational satisfaction, efficiency, effectiveness and organizational productivity, nurses’ health and their professional relationships, identity and self-esteem; other outcomes may include creation of a favorable and growing organizational climate, enhancement of patient safety, and a decrease in occupational burnout and quitting. Eventually, all these factors would bring about an overall improvement of the quality of care, which is the end goal of every health system (13, 14, 29,34). We hope that the results of the present study will improve the performance of clinical nurse managers in decision-making and direct their actions and managerial activities toward moral decision-making.

In fact, picturing the lived experiences of clinical nurse managers in their occupational context can help their managerial group resolve the moderator obstacles to moral distress. As a result, they will be able to make more appropriate moral decisions and decrease their moral distress. The most important product of phenomenology is gaining insight into a phenomenon, which adds to the nursing profession's knowledge in this field. Considering that ethical decision-making by nurse managers is the most important aspect that affects the overall quality of nursing care, this insight can increase nurse managers' ability to fulfill their management role in a more effective, holistic and comprehensive manner by enhancing the power of moral reasoning.

## Method

The present study was based on qualitative research. The author used hermeneutic phenomenology to find an appropriate answer to the research question about clinical nurse managers’ lived experiences of moral distress.

Participants in the present study were 14 clinical nurse managers including matrons, supervisors and head nurses. Participants had at least five years of nursing management experience in hospitals, and therefore boasted rich experiences of the subject. 

The study was performed in places where the participants lived their professional lives as clinical nurse managers. Interviews were conducted in the teaching hospitals affiliated with Tehran University of Medical Sciences.

In the present study, the main method of data collection was in-depth semi-structured personal face-to-face interviews. Interviews lasted from 40 to 75 minutes. Fourteen participants (six men and eight women) were selected based on the mentioned criteria and using purposeful sampling method. The effort was to select participants with maximum diversity. The topic of the study was rather subjective and required pre-contemplation on the part of the participants to understand and remember the experiences. Accordingly, the researcher visited the study environment one week before every interview and gave the prepared narrative paper to the participants to ensure their understanding of the study topic. This paper included the title of the study, its aim, and definitions of the words and concepts. Also, it requested the participants to explain one of their experiences of moral distress in detail. On the day of the interview, the completed papers were gathered so that a comprehensive analysis could be performed on the data at the same time the data transcription took place. Interviews started with open questions such as “What comes to your mind when you hear the term ‘moral distress’?” or “What does moral distress mean to you?” Then the interviews continued with more focused questions in line with the topic of the research, which was the nature of clinical nurse managers’ lived experiences of moral distress. Four of the participants needed to be interviewed twice to clarify the ambiguous points in their first interviews.

**Data Analysis**

Data analysis was performed in line with the aim and method of the study using the Diekelmann, Allen and Tanner method (1989), which is a seven-stage process based on hermeneutic phenomenology (35). Accordingly, the interviews were transcribed verbatim after being repeatedly reviewed on the same day. The transcriptions were reexamined several times to gain a comprehensive understanding of the data. Subsequently, key phrases in the texts were marked as the meaning units. At the next stage, the entire texts of the interviews and narrative papers were reviewed and the interpretative summary was written. Considering the emphasis of phenomenology on discovering the meanings of phenomena, the researcher’s effort was to search for and extract all the main and sub-themes during the data analysis process based on the “lived experiences of clinical nurse managers regarding moral distress.” To explain, categorize and resolve any disagreements and conflicts in the interpretations, the process of referring to the texts or the participants was regularly performed through phone calls and direct contacts. The process of consensus of the ideas and extracted concepts led to the formation of subcategories. During the next steps, and by eliminating similar or overlapping and merging options, the subcategories became more comprehensive and the main themes were formed.

**Ethical Considerations**

The study protocol was approved by the IRB of the Tehran University of Medical Sciences; register number 9323199001. We provided the necessary information about the goals and method of the study to the potential participants and obtained their written informed consent at that time. The participants also provided permission for recording the interviews; moreover, they were reminded of their right to withdraw from the study at any time and were assured that their confidentiality, including anonymity and data protection, was valued. 

**Study Rigor**

Concepts of credibility, confirmability, auditability and transferability were used for maintaining the rigor of the study (35, 37). Credibility of the study was evaluated by keeping a prolonged engagement with the data (more than 12 months), and employing the member and peer checking techniques. After the analysis, the participants were contacted and given a full transcript of their respective coded interviews with a summary of the emergent categories to approve the interpretations of the researchers. The study process was checked by expert supervisors and two doctoral students of nursing, and all evidence and documents were saved securely to maintain auditability. Moreover, an adequate thick description of the context was carried out so that a judgment of transferability could be made by the readers.

## Results

The findings related to nurse managers’ experiences of clinical moral distress contained two main themes and four sub-themes. These themes were the result of 18 interviews with 14 participants. The main themes were psycho-emotional trauma and professional desperation syndrome. Psycho-emotional trauma contained the subthemes of shame and emotional dissociation. The second main theme was professional desperation syndrome, with the subthemes of helplessness and impaired professional identity.

Table 1 presents the demographic characteristics of the participants, and the themes, subthemes and their meaning units are shown in Table 2.

**Table 1:** A summary of the participants’ demographic characteristics

Number	Gender	Age	Marital Status	Education Level	Years of Service	Hospital	Managerial Position	Interview Duration (minutes)
1	Female	45	Married	BS	20	Public	Matron	65
2	Male	28	Single	BS	5	Public	Supervisor	70
3	Male	31	Married	MS	10	Private	Head Nurse	50
4	Female	51	Married	BS	25	Public	Matron	55
5	Female	30	Married	MS	6	Private	Supervisor	60
6	Female	32	Single	BS	5	Private	Supervisor	45
7	Female	27	Married	BS	5	Public	Head Nurse	55
8	Male	33	Married	BS	10	Public	Head Nurse	40
9	Female	26	Married	MS	7	Public	Head Nurse	45
10	Male	40	Married	BS	18	Private	Matron	45
11	Female	25	Married	BS	9	Public	Supervisor	50
12	Female	34	Single	PhD	10	Private	Head Nurse	45
13	Male	39	Married	PHD	13	Private	Supervisor	75
14	Male	35	Married	MS	10	Public	Matron	60

**Table 2:** Achieved results from the participants’ experiences

Main Theme	Subtheme	Meaning Units
Psycho- Emotional Trauma	Shame	Words sticking in one’s throat - being crushed under the hidden mental pressure - endurance of nervous pressure - being stuck in a weak position – feeling heaviness in the heart - self-blame - feeling ashamed - The tendency to disguise oneself - regret and remorse - feeling sorrow and hatred
	Emotional Dissociation	Having a breakdown - collapsing - conflict- contradiction and duality – inconsistency, struggle and contradiction between learning and performance – a sense of paradox - emotional destruction
Professional Desperation Syndrome	Helplessness	Loneliness - the sense of being in a black hole - isolation - self-absorption – depression - lack of motivation - unpleasant feeling of doing the wrong thing - sadness and sorrow associated with wrong decisions - spiritual suffering – mental and spiritual disorganization - mental disorder and disturbance - fear and discomfort - mental destruction - distress - exhaustion - stress - concern – anxiety - the sense of burnout - lack of energy while working – disappointment - intellectual inability and disability
	Impaired Professional Identity	Lack of courage to perform one’s duties - decline of professional relationships - stepping out of the professional mission - - impaired occupational integrity - professional incompetency - feeling of incapability – professional irresponsibility - decreased professional self-esteem - disruption of teamwork - professional dishonesty

*Main Themes:*

- *Psycho-Emotional Trauma:*

Psycho-emotional trauma was one of the main themes of moral distress among clinical nurse managers, and contained the subthemes “shame” and “emotional dissociation.” This theme indicated that clinical nurse managers’ experiences of moral distress had been associated with resentment, self-blame, and lack of peace of mind. Furthermore, some of the managers mentioned a kind of moping and embarrassment that accompanied them at all times. They also stated that tolerating the pressure for a long period would lead to breakdown and collapse.

   a)     Shame

The first subtheme was shame, which is defined as feeling embarrassed or guilty because of one's actions, characteristics, or associations. In this regard, nurse managers from various levels stated that when an individual knows right and wrong but is forced to do the wrong thing for organizational reasons or on account of the work environment and lack of supportive laws, he or she will experience incomprehensible and constant pressure. It feels like constant self-blame and embarrassment, and will interrupt one’s professional performance.

In this regard one of the managers stated:

*“For example, I may have a good opinion of an* *employee and believe that he or she has an appropriate professional behavior, but when that person is punished by one of the supervisors, even though I know the employee is right, I cannot defend him or her and have to stay quiet. This will make me blame myself constantly. I feel stuck in a weak position and under great pressure.”* [Participant No. 3]

Similarly, another participant stated:

*“On one of my work days, the ambulance brought in a 70-year-old man and immediately started CPR on him by order of the emergency physician who was also one of the shareholders of the hospital. After the patient died, the physician ordered his secretary to admit him in the critical care unit. I could not refuse the orders because if I did, I would be fired. Whenever I think about it, I feel like something is stuck in my throat that I cannot get rid of, and I can’t speak to anyone about it either…” *[Participant No. 5]

   b)     Emotional Dissociation

One of the extracted subthemes confirmed by the participants was “emotional dissociation”, which is a feeling of isolation, emotional management problems, sudden and unexpected conflicts, depression or anxiety. Participants expressed their general understanding and impression of the experienced moral distress through terms such as breakdown, collapse, conflict, contradiction and so on.

*“In the nursing profession, every day is filled with distress. For example, a physician visits a patient every day, and I know that the patient does not need daily visits, but the physician does it for financial reasons; I cannot object to this behavior because the entire system is working this way. This makes me break down silently every day from the inside”* [Participant No. 11]

Another participant mentioned that their experience of distress was a constant conflict and contradiction between learning and performance and stated:

*“I have been the head nurse for a long time. Sometimes we have patients who should undergo chemotherapy, and you know that chemotherapy drugs should be prepared in the incubator and under the fume hood. I know that without the necessary equipment, this procedure could threaten my nurses’ health, but I ask them to do it anyway. I know that it is completely wrong, but there is no other way. I have talked about this to the higher managers several times, and they have responded that we cannot afford the costs for a couple chemotherapy patients a month, and I cannot stop the treatment. But when I see the blisters caused by drug preparation on the nurses’ hands, I feel distressed and conflicted for several days”* [Participant No. 9]

- *Professional Desperation Syndrome:*

Another main theme that emerged was “professional desperation syndrome”, which is a feeling of despair and hopelessness, and typically one that results in a certain behavior. This main theme contained the subthemes of “helplessness” and “impaired professional identity”. It is clear that desperation at exhibiting professional behavior has become a disturbing issue in the nursing profession.

   a)     Helplessness

Helplessness, which is a feeling or state of despair, or lack of hope, is one of the subthemes of desperation in performing professional behavior. Participants used the following phrases to describe their experiences of moral distress: loneliness, the sense of being in a black hole, isolation, self-absorption, and depression.

*“I was a manager in a center for lung transplant. The night before this particular case of transplant, a nurse from the medical human resources was assigned to the patient, and the physician went home. The charge nurse also took the day off, so a nurse with one year of work experience stayed with the patient. When we arrived at the center in the morning, the patient was deceased. While I was writing a report for the forensics office, I was stopped by the* *Chief executive officer of the hospital, who was one of the friends of the physician in charge. There, I felt the worse kind of mental disturbance. I was filled with distress and desperation. I will never forget that day. Even as I think about it now, I feel completely drained” [*Participant No. 14]

Another participant also described their experiences of moral distress with concepts such as exhaustion and disability:

*“Occasionally, personnel should perform a procedure for the patient, such as dressing. But they did not have. You know that they overlooked it and performed the wrong action, so you should be on the patient’s side. On the other hand, if you say this to the patient, there will be trouble. The complaints and the [possible] refusal to pay the costs… it can get complicated. It might even cause the personnel to get fired. In such situations, I feel worried and mentally disturbed. My brain stops working, and I feel disabled for not reporting the incident”* [Participant No. 9]

   b)     Impaired Professional Identity

Impaired professional identity means diminished professional self-concept based on one’s attributes, beliefs, values, motivations and experiences, and was the second subtheme of desperation in performing professional behaviors. 

Regarding their experiences of moral distress, one of the participants said:

*“Sometimes it happens that the patient does not need hospitalization, but has been hospitalized in the ward for a long time by the physician’s order; or the patient receives daily visits while we know that these visits are not necessary. We just have to close our eyes and ignore these things and not report them. Here, we think we are incompetent in our profession, because we feel incapable and dishonest. This feeling eats you from the inside and will get worse every time you confront such situations”* [Participant No. 4]

Another participant said about their experiences:

*“There was once this person who applied for a job with us, but had not completed the medical human resources program and was still a student. We were not legally allowed to hire that person, but you know, due to a shortage in the nursing staff, we hire whoever comes for employment, unless they are really not qualified. Whenever I hire someone like this, I feel irresponsible toward my patients. These issues cause distress and exhaustion in me. But now I am used to the feeling, because I have no other option”* [Participant No. 10]

## Discussion

In order to understand the phenomenon of moral distress among nurse managers, it is necessary to investigate the moral distress experienced by clinical nurse managers. Although clinical nurse managers repeatedly encounter moral distress in their daily decisions and duties, they are not fully aware of the phenomenon (19). An examination of clinical managers’ experiences and perceptions of moral distress led to the extraction of two main themes: “psycho-emotional trauma” and “professional desperation syndrome”. The main theme of psycho-emotional trauma contained two subthemes of “shame” and “emotional dissociation”. The main theme of professional desperation also contained subthemes of “helplessness” and “impaired professional identity”. These themes and their related subthemes are new concepts that are presented in this study.

*The first main theme of the present study:* As one of the main themes in the present study, “psycho-emotional trauma” has provided a clear image of nurse managers’ experiences of moral distress in their profession. The subthemes of “shame” and “emotional dissociation” are correlated with each other and with the main theme, and certainly confirm it. The term “psycho-emotional trauma” was selected due to the mental and emotional damage that nurse managers endure upon experiencing moral distress. Psycho-emotional trauma may not only lead to burnout in managers, but it can also cause them to lose motivation and quit their job.

The first subtheme of psycho-emotional trauma was “shame”. In their study “Moral Reckoning in Nursing”, Nathaniel stated that nurses who are forced to take the wrong action despite their knowledge and awareness will experience self-blame, shame, embarrassment and regret (6). The results of their study are rather similar to the present study and indicate pressure on nurses who experience moral distress. In both of these studies, nurses who considered themselves responsible for an unethical act suffered from constant self-blame. This would cause mental and emotional damage in nurses. Austin et al. also mentioned in their study that such experiences were associated with psycho-emotional symptoms such as disappointment, feelings of worthlessness, shame and self-blame in nurses (4). In this regard, the results of a study by Kelly showed that when nurses acted against their interests and beliefs, they experienced moral distress, followed by moral judgment. The latter would cause individuals to constantly blame themselves for knowingly committing an immoral act, and thus feel ashamed. This constant feeling of shame would make the nurses feel disappointed, which would in turn cause them to suffer from mental and emotional distress (38).

The second subtheme was “emotional dissociation". This subtheme entails conflict and contradiction. Clinical managers described their experiences of moral distress using words and phrases such as breaking down, collapsing, conflict, contradiction and duality, inconsistency and conflict between learning and performance, paradox, and emotional destruction. When clinical nurse managers know what the right act or decision is but they are forced to commit an immoral act, their emotional integrity is damaged and they may consider quitting the nursing profession. In fact, the sense of conflict and paradox is like an alarm for managers that something is wrong. Hendel et al. has defined conflict as an internal inconsistency rooted in thoughts, values or emotions (39). In a qualitative study on moral distress conducted in 2016, Dzeng et al. mentioned that the differences between individuals’ internal moral values and their professional organizational values and principles would push them toward emotional dissociation. The conditions that cause conflict and emotional dissociation could create the grounds for negative outcomes that might affect the health and medical systems (40). Henrich et al. conducted a qualitative study in 2017 titled “Consequences of Moral Distress in Intensive Care Units: A Qualitative Study.” In line with the results of the present study, they stated that nurses would suffer from negative emotional conflicts when they experienced moral distress. They also found that the conflict and inconsistency between nurses’ learning and performance affect the quality of the care they provided (41).

*The second main theme of the present study:* The second main theme was “professional desperation syndrome”, which contained two subthemes of “helplessness” and “impaired professional identity”. Christodoulou-Fella et al. and Meltzer and Huckabay conducted a study about the factors influencing burnout and exhaustion in nurses as the main reasons for quitting the job. They stated that stressful work environment, moral or emotional distress, mental and emotional disturbance, exhaustion, lack of motivation, lack of professional self-esteem, inability, doubt, ambiguity and confusion were the main reasons for burnout and quitting the job in nurses. The above-mentioned reasons are in consistence with clinical nurse managers’ experiences of moral distress. In total, they led to the main theme of “professional desperation syndrome”, which contains various aspects that are mentioned in the results (42, 43).

Regarding the subtheme of “helplessness”, results of a study by Wilkinson is in line with those of the present study. They found that at various levels of nursing, the experience of moral distress was generally associated with sleep problems, anger, disappointment, discomfort and lack of energy, and would also cause severe helplessness in the individual. When lasting for a long period of time, this sense of helplessness could lead to professional burnout, and would affect the quality of care directly and indirectly (33). Similarly, a study by Abbaszadeh et al. revealed that morality is an inseparable part of clinical performance, and professional helplessness is always associated with moral distress (45). In a study by Joolaee et al., 41% of the nurses stated that they would not choose this profession again. They also stated that they were feeling powerless, disabled, disappointed and professionally burnt-out due to the moral distress they were experiencing in the work environment (46). Flinkman et al. also conducted a study about professional helplessness in nurses and quitting the job, which showed that one of the main reasons for nurses to quit their job and change their work shift was the sense of helplessness. The feeling of helplessness and disability caused by long-term occupational and moral distress in nurses would lead to burnout and decrease the quality of care (47).

Regarding the subtheme of “impaired professional identity”, Harrowing and Mill found that in nursing, professional identity and integrity are influenced by moral factors, which is confirmed by the results of the present study. They also stated that experiencing moral distress would affect the professional identity of nurses (48). In a qualitative study on disrupted professional identity, Baraz-Pordanjani et al. mentioned that working in an environment where individuals felt impaired identity, professional incompetence and decreased self-esteem could severely disrupt their professional identity. Therefore, it may be concluded that moral distress and organizational pressures could disrupt professional identity and even the quality of nursing care (49). Another qualitative study that confirms our findings was conducted by Vaismoradi et al., and examined factors affecting the formation of professional identity in nurses, for instance the existing gap and inconsistency between learning and performance, disrupted professional relationships, and work environment moral distress. Moreover, work environment and perceived moral distress by the nurses are considered as factors that help shape nurses’ professional identity (50).

Finally, it should be mentioned that in comparison with previously existing research, the present study presented a deeper description and interpretation of clinical nurse managers’ perception of moral distress. Understanding these experiences will be an introduction to modifying the associated negative outcomes in the future, and creating a suitable environment for moral decision-making. Therefore, recognizing moral distress in clinical managers can improve their planning and management skills in dealing with it, and this will be of great help to the healthcare system of any country.

## Conclusion

Moral distress is an important phenomenon in nursing management that affects the body and soul of managers. The present study explored moral distress based on Iranian nurse managers’ lived experiences. For clinical nurse managers, moral distress is an ambiguous situation like suspension between the earth and the sky in uncertainty and fear, and may be a reaction to having to knowingly cover up intentional and unintentional mistakes. The experience is like a hidden suffering and spiritual torment caused by betraying moral principles. In the experience of clinical managers, moral distress is equal to confusion, anxiety and concern about the outcomes. Not only would it cause unbearable mental suffering and self-blame, but it could also lead to resignation and quitting the managerial position. The managers stated that it seemed as if their organizational commitments had priority over their human obligations. They believed that experiencing this kind of conscious mistake was the reason for the occurrence of professional desperation syndrome and psycho-emotional trauma.
